# Dopexamine can attenuate the inflammatory response and protect against organ injury in the absence of significant effects on hemodynamics or regional microvascular flow

**DOI:** 10.1186/cc12585

**Published:** 2013-03-28

**Authors:** Mansoor N Bangash, Nimesh SA Patel, Elisa Benetti, Massimo Collino, Charles J Hinds, Christoph Thiemermann, Rupert M Pearse

**Affiliations:** 1 Department of Translational Medicine and Therapeutics, The William Harvey Research Institute (Queen Mary's School of Medicine and Dentistry), John Vane Building, Charterhouse Square, London EC1M 6BQ, UK; 2 Department of Drug Science and Technology, University of Turin, via Pietro Giuria 9, 10125 Torino, Italy; 3 Adult Critical Care Unit, Royal London Hospital, London E1 1BB, UK

## Abstract

**Introduction:**

The effects of dopexamine, a β2-agonist, on perioperative and sepsis-related hemodynamic, microvascular, immune, and organ dysfunction are controversial and poorly understood. We investigated these effects in a rodent model of laparotomy and endotoxemia.

**Methods:**

In two experiments, 80 male Wistar rats underwent laparotomy. In 64 rats, this was followed by administration of endotoxin; the remainder (16) underwent sham endotoxemia. Endotoxemic animals received either dopexamine at 0.5, 1, or 2 μg/kg/min or 0.9% saline vehicle (controls) as resuscitation fluid. The effects of dopexamine on global hemodynamics, mesenteric regional microvascular flow, renal and hepatic function and immune activation were evaluated.

**Results:**

Endotoxin administration was associated with a systemic inflammatory response (increased plasma levels of tumor necrosis factor (TNF)-α, interleukin (IL)-1β, IL-6, and IL-10, as well as cell-adhesion molecules CD11a and CD11b), and increased pulmonary myeloperoxidase (MPO) activity (indicating pulmonary leukocyte infiltration), whereas biochemical changes demonstrated lactic acidosis with significant renal and hepatic injury. Dopexamine administration was associated with less-severe lactic acidosis (pooled dopexamine versus controls, (lactate, 2.2 m*M *± 0.2 m*M *versus 4.0 m*M *± 0.5 m*M*; *P *< 0.001) and reductions in the systemic inflammatory response (pooled dopexamine versus control, 4 hour (TNF-α): 324 pg/ml ± 93 pg/ml versus 97 pg/ml ± 14 pg/ml, p < 0.01), pulmonary myeloperoxidase (MPO) activity, and hepatic and renal injury (pooled dopexamine versus control (ALT): 81 IU/L ± 4 IU/L versus 138 IU/L ± 25 IU/L; *P *< 0.05; (creatinine): 49.4 μ*M *± 3.9 μ*M *versus 76.2 μ*M *± 9.8 μ*M*; *P *< 0.005). However, in this study, clinically relevant doses of dopexamine were not associated with clinically significant changes in MAP, CI, or gut regional microvascular flow.

**Conclusions:**

In this model, dopexamine can attenuate the systemic inflammatory response, reduce tissue leukocyte infiltration, and protect against organ injury at doses that do not alter global hemodynamics or regional microvascular flow. These findings suggest that immunomodulatory effects of catecholamines may be clinically significant when used in critically ill surgical patients and are independent of their hemodynamic actions.

## Introduction

A growing body of evidence suggests that the potential exists to reduce the morbidity and unacceptably high mortality rates associated with major surgery in high-risk patients [1]. For many years, inotropic and vasoactive agents have been widely used to maintain tissue perfusion in critically ill and high-risk surgical patients with the aim of improving clinical outcomes [2]. Dopexamine is a dopamine analogue with agonist activity at β2 and dopaminergic receptors. This spectrum of activity confers vasodilator actions in addition to chronotropic and mild inotropic effects. Postoperative complications occur more frequently in the presence of poor tissue microvascular flow and oxygenation [3-5], and dopexamine has been shown to improve these abnormalities [6]. However, the effect of dopexamine on clinical outcomes is less clear, and the findings of randomized trials have proved inconsistent [6-11]. These conflicting findings might be explained by dose-related differences in the hemodynamic and immunologic effects of dopexamine [12].

Increasing recognition is building that adrenergic agents may have important metabolic and immunologic actions [13,14], whereas tachycardia and myocardial ischemia may cause significant harm, especially at higher doses. It has been suggested that anti-inflammatory actions may be beneficial [13,15]. Previous work indicated that dopexamine may decrease leukocyte-endothelial adhesion in mesenteric venules [16,17], a phenomenon dependent on CD11a and CD11b integrins [18]. Other adrenergic agents have also been shown to exert antiinflammatory actions on cytokine responses in immune cells [15,19,20]. It is possible that the proposed clinical benefits of dopexamine, particularly at low doses, may relate to actions on inflammatory pathways [21]. Our objective was to investigate the effects of dopexamine on global hemodynamics, regional microvascular flow, systemic inflammatory response, and organ injury in a rodent model of laparotomy and endotoxemia.

## Materials and methods

Eighty male Wistar rats (220 to 410 g) received a standard diet and water *ad libitum *before experiments (two experiments used 40 rats divided into five groups and shared a common anesthetic and early surgical procedure, as detailed later). All procedures were performed with institutional approval and in accordance with the Home Office Guidance on the Operation of Animals (Scientific Procedures) Act, 1986. Anaesthesia was induced by intraperitoneal injection of thiopental (120 mg/kg) and maintained with supplementary injections administered according to regular testing for limb withdrawal to a standard stimulus or signs of inadequate anaesthesia. Animals were placed on a heated mat and maintained at 37 ± 0.5°C. A tracheostomy was performed, following which a short section of polyethylene tubing (internal diameter, 1.67 mm) was inserted to maintain airway patency and to facilitate spontaneous respiration. The right carotid artery was cannulated to allow blood sampling and continuous monitoring of heart rate (HR) and mean arterial pressure (MAP). The left jugular vein was cannulated for drug and fluid administration. A 2-cm midline incision was then made through the abdominal wall to expose the small intestine. This completed the shared early surgical procedure (further surgery was performed, as specified in later sections for each of the two experiments). After the completion of surgery and stabilization in both experiments, this protocol was followed: endotoxemia was induced in four of five groups by administration of *Escherichia coli *lipopolysaccharide (LPS) 0111:B4 (6 mg/kg) over a 10-minute period (sham group received 0.9% saline vehicle). Administration of LPS was followed by 4 hours of fluid resuscitation with an infusion of intravenous 0.9% saline at 4.3 ml/kg/h. Sham and control animals received only 0.9% saline infusion. Three different concentrations of dopexamine were added to the remaining three groups' infusion fluid, producing dopexamine infusion rates of 0.5, 1, and 2 μg/kg/min for groups D0.5, D1, and D2, respectively. The experiment ended after 4 hours of resuscitation when the lungs and heart were harvested *en bloc*. In experiment 1, the lungs were flash frozen in liquid nitrogen before being stored at -80°C for subsequent analysis.

### Experiment 1

In the first set of 40 rats, after the early shared surgical procedure, a loop of intestine adjoining the terminal ileum was exteriorized and placed in a Saran receiving pouch. A baseline set of arterial blood samples was then taken, the volume being replaced with an equal volume of 0.9% saline. Animals were allowed to stabilize for 15 minutes before being randomized to one of five groups: sham, control, D0.5, D1, or D2. Each group contained eight animals, and all animals underwent surgery and resuscitation. Only sham animals did not receive LPS, whereas neither sham nor control animals received dopexamine.

#### Measurement of plasma lactate, base deficit, and indices of renal and hepatic injury

A 200-μl blood sample was taken at baseline and at the end of the experiment for measurement of plasma lactate concentration (Accutrend Lactate; Roche Diagnostics, Basel, Switzerland) and base deficit (Radiometer, Copenhagen, Denmark). A 1-ml blood sample was also taken at the end of experiment for the measurements of urea, creatinine, alanine aminotransferase (ALT), and aspartate aminotransferase (AST) by a commercial veterinary laboratory (IDEXX Laboratories Ltd, Sussex, UK), who were blinded to treatment.

#### Measurement of plasma cytokine levels

A 200 μl blood sample was taken for measurement of plasma cytokine levels at baseline, 60 minutes after LPS administration, and at the end of the experiment. Samples were centrifuged immediately at 9,900 *g *for 3 minutes. A minimum of 50 μl of plasma per sample was collected and stored at -80°C for subsequent analysis. Cytokine levels were measured on a Luminex 200 reader (Luminex Co., Austin, TX, USA) by using the Rat Cytokine 10-Plex kit (Invitrogen Corporation, Camarillo, CA, USA) and following manufacturer's instructions. Measurements were expressed as mean fluorescent intensity, which was converted to pictograms per milliliter by using a set of nonlinear transforms based on standard curves created in PrismGraph 4.0 (GraphPad Software Inc., San Diego, CA, USA).

#### Measurement of pulmonary myeloperoxidase levels

MPO was measured in samples of pulmonary tissue from lungs harvested at the end of the experiment. These samples were stored at -80°C and were analyzed in four randomly selected lung samples per group by colleagues who were blinded to treatment. Samples of tissue from the right lung were homogenized in a solution containing 0.5% (wt/vol) hexadecyltrimethyl-ammonium bromide dissolved in 10 m*M *potassium phosphate buffer (pH 7) and centrifuged for 30 minutes at 20,000 *g *at 4°C. An aliquot of the supernatant was then allowed to react with a solution of 1.6 m*M *tetramethylbenzidine and 0.1 m*M *H_2_O_2_. The rate of change in absorbance was measured spectrophotometrically at 650 nm. MPO activity was defined as the quantity of enzyme degrading 1 μmol of peroxide per minute at 37°C and was expressed in microunits per gram wet tissue.

#### Measurement of neutrophil cell surface expression of CD11a and CD11b

Phycoerythrin and fluorescein isothiocyanate-conjugated mouse monoclonal antibodies (mAbs) against CD11a (IgG2a) and CD11b (IgA) were used to quantify neutrophil cell-surface expression of these markers. Isotype-, fluorochrome-, and protein concentration-matched controls were run in parallel to the mAb (Becton Dickinson, Oxford, UK). Heparinized blood (600 μl) was collected for flow-cytometric analysis of leukocyte adhesion molecules, and 100 μl of whole blood was mixed with mAb against rat CD11a (5 μl) or CD11b (3 μl) in 4 × 75-mm polystyrene test tubes. Blood with no antibody added served as a control for autofluorescence. The test tubes were then incubated on ice for 30 minutes with continuous shaking, protected from light. Erythrocytes were lysed by addition of 2 ml FACSTM lysing solution to the test tubes. The samples were then incubated for a further 10 minutes on ice in the dark, and then centrifuged at 1,000 *g *for 3 minutes at 4°C. The supernatant was discarded, and the leukocyte pellet was resuspended and washed twice in 2 ml ice-cold optimized PBS cell wash. Finally, leukocytes were fixed in 0.3 ml 1% wt/vol paraformaldehyde in PBS at pH 7.4, and the tubes were stored in the dark at 4°C for up to 24 hours until flow-cytometric analysis could be performed. Samples were analyzed by using a FACScan flow cytometer equipped with Cell-Quest software. CaliBRITE-3 beads and FACS COMP software were used on a weekly basis to calibrate the fluorescence intensity in accordance with the manufacturer's instructions. Ten thousand neutrophils were collected from each sample with light-scatter gain set in the linear mode and fluorescence gain set in the logarithmic mode. The neutrophil population was identified by light-scatter characteristics (forward versus side-scatter) and enclosed in an electronic gate for fluorescence histogram analysis. Antibody binding was expressed as mean fluorescence intensity (MFI), values for which were corrected for nonspecific binding by subtracting MFI measured for the matched isotype control sample.

### Experiment 2

Subsequent to the findings of the first experiment, a second experiment using 40 rats (eight animals randomized to five groups, as in experiment 1) was performed to determine the effects of the three doses of dopexamine on cardiac output and mesenteric regional microvascular flow. The same protocol was followed, as specified previously, except that, after laparotomy, bowel was evacuated into a moist cotton receptacle. Blunt dissection was then performed to access the abdominal vasculature. After isolation from the vena cava, a 1.5-mm ultrasonic aortic transit time flow probe (MA1.5PRB; Transonic Systems Inc., Ithaca, NY, USA) was placed on the infrarenal aorta to measure aortic blood flow. The bowel was then replaced in the abdominal cavity, except for a loop of ileum just proximal to the cecum. The exposed bowel was kept moist by the application of 0.9% saline drops through a pipette. The laparotomy incision above and below the exit of the terminal ileal loop from the abdomen was then closed with 5.0 Vicryl to prevent excessive insensible losses. A 1.5-cm incision was subsequently made in the antemesenteric border of the ileum by using unipolar diathermy for later placement of laser Doppler probes. To prevent thermal damage to the ileum, the section was immediately washed with normal saline. The mucosal surface of the bowel was exposed and gently cleansed with 0.9% saline by using cotton-tipped buds in preparation for placement of laser Doppler probes. The animal was then placed on a Perspex stage in the right lateral position so that the ileal loop rested on a raised section of the stage, at the level of the laparotomy incision. Subsequently the bowel was fixed at two points on either side of the incision with a small amount of tissue glue to prevent movement artefact. After application of the laser Doppler probes (see later), an impermeable cover for the ileal loop was created by placing small pieces of pre-cut Saran wrap over the loop and around the probes, until the ileal preparation was airtight. This was followed by a 5-ml/kg bolus of intravenous normal saline to replace insensible fluid losses and by a 15-minute stabilization period. Blood sampling for plasma lactate and arterial blood gases was performed, followed by a second 15-minute stabilization period before measurement of regional microvascular flow. The only other blood samples taken were at the end of the experiment for markers of organ injury.

#### Measurement of mesenteric red cell flux

Two fiberoptic laser Doppler flux slave probes (P10k; Moor Instruments, Axminster, UK) suspended from clamps were lightly applied to the mucosal and serosal surfaces of the ileum to determine red-cell flux, a measure of regional microvascular blood flow. Two probes were placed on the ileum, one on a mucosal site and one on a serosal site away from visible blood vessels. These were then fixed with tissue glue, a technique that causes minimal interference with tissue microvascular flow. Slave probes were calibrated daily by using PFS flux standard (Moor Instruments, Axminster, UK) at 23°C before experiments. Slave probes were attached to master probes (MP10M200ST; Moor Instruments), which in turn were connected to a satellite monitor (moorLAB; Moor Instruments). Connection of the server to a desktop computer allowed continuous recording of red-cell flux and the direct current or DC signal (index of reflected light intensity and hence quality of probe contact). Laser light of 780-nm wavelength with a 40-Hz sampling rate and a 30-degree angular spread allowed a sampling volume of approximately 1 mm3. The probe readout was monitored for 2 minutes to ensure adequate contact before fixation. As the thickness of rat ileum is less than the depth of measurement achieved with LDF probes, red-cell flux was averaged between mucosal and serosal sites to minimize bias due to heterogeneity in regional microvascular flow.

#### Measurement of aortic blood flow

A 1.5-mm perivascular probe was applied with water-soluble sonicating gel and sited as described earlier. The probe was connected to a TS420 monitor (Transonic Systems Inc., Ithaca, NY, USA), which was connected to a Powerlab/8SP monitoring system (AD Instruments). This allowed continuous recording of aortic blood flow and waveform-derived HR and calculation of a measure of stroke volume. Aortic blood flow was indexed to body weight to provide a measure of changes in stroke volume index (SVI) and cardiac index (CI). Probe calibration was performed daily according to the manufacturer's instructions before experiments.

### Statistical analysis

In both experiments, D'Agostino & Pearson Omnibus normality testing was performed on all data (Kolmogorov-Smirnov testing if missing data points meant numbers in the group were too small for this). Normally distributed data was tested by using one-way analysis of variance (ANOVA) for comparison across all groups at a given time point, and two-way ANOVA, for changes in multiple groups over time (that is, repeated measurements). Posttesting was performed with Bonferroni tests. When data were not normally distributed in at least one group for any measurement (for example, urea, experiment 2), the Kruskal-Wallis test was used in place of one-way ANOVA, and appropriate *t *tests against controls for posttesting were used, depending on whether the individual groups were normally distributed.

In each experiment, when no significant differences were found between dopexamine groups, data were pooled and compared against the corresponding control group by using two-tailed unpaired *t *tests (normally distributed) or Mann-Whitney tests otherwise. This occurred in the case of base deficit, lactate, and organ function. Two-tailed paired *t *tests were used to compare hemodynamics and microvascular flow at baseline with end of experiment for animals within the same group. Data were analyzed with PrismGraph 4.0 (GraphPad Software, San Diego, CA, USA). Significance was set at *P *< 0.05.

## Results

Of a total of 3,740 measurements across the two experiments, 33 either were missed because of equipment problems or were inaccurate. The numbers of absent data points are referred to in the figure legends, and were mostly related to measurements of serum base deficit, lactate, cytokines, and integrin expression.

### Experiment 1

**Figure 1:**
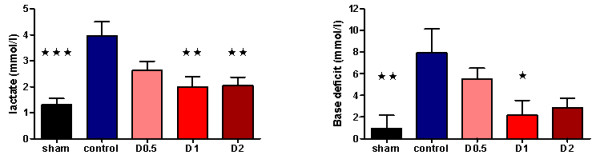
**Plasma lactate (*n *= 8 for all groups) and base deficit (*n *= 8 all except D0.5 and D1 (*n *= 6 each)) at 4 hours after laparotomy and endotoxemia, experiment 1**. In control animals, lactate significantly increased compared with shams, whereas this was not observed in dopexamine-treated animals (*P *< 0.001 pooled dopexamine versus control). A similar observation was made for base deficit (*P *< 0.05 pooled dopexamine versus control). Data presented as mean (SEM). One-way ANOVA (Bonferroni posttests, *P < 0.05, **P < 0.01, ***P < 0.001 compared with controls).

**Figure 2:**
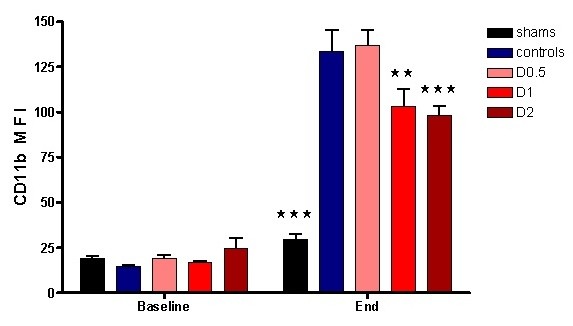
**Circulating neutrophil surface CD11b mean fluorescent intensity (MFI) at baseline and 4 hours after laparotomy and endotoxemia (*n *= 6 controls, *n *= 8 all others), experiment 1**. No significant differences were found between groups at baseline. Dopexamine was associated with smaller increases in D1 and D2 groups compared with controls. Data presented as mean (SEM). Two-way ANOVA (Bonferroni posttests, **P < 0.01, ***P < 0.001 compared with controls).

**Figure 3:**
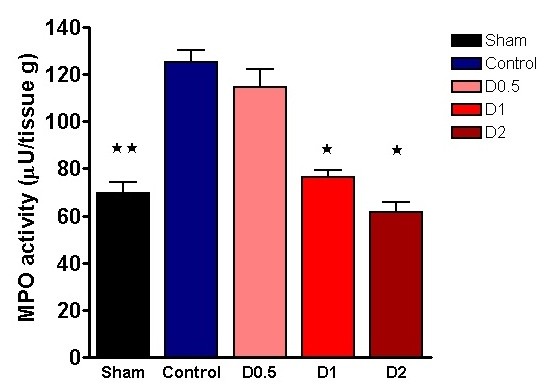
**Pulmonary myeloperoxidase (MPO) activity in lung tissue sampled 4 hours after laparotomy and endotoxemia (*n *= 4 each group), experiment 1**. Dopexamine was associated with smaller increases in D1 and D2 groups compared with controls. Data presented as mean (SEM). One-way ANOVA (Bonferroni posttests, *P < 0.05, **P < 0.01 compared with controls).

**Figure 4:**
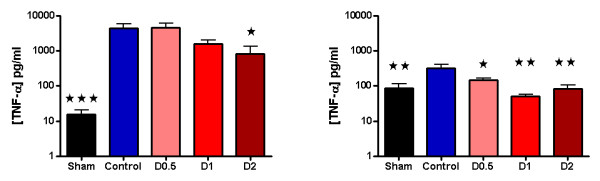
**One-hour (peak) plasma TNF-α concentrations (*n *= 8 except controls and D2 (*n *= 7 each)) and 4 hours (*n *= 8 except D0.5, D1, and D2 (*n *= 7, 6, and 5, respectively)) wing laparotomy and endotoxemia, experiment 1**. Dopexamine was associated with smaller increases in all groups at 4 hours, and in D2 at 1 hour, compared with controls. Data are presented as mean (SEM) for clarity at both time points. However, not all data from 1 hour were normally distributed; the statistical analysis reflects this. One hour (t1): Kruskal-Wallis test (*post hoc *Mann-Whitney tests, *P < 0.05, ***P < 0.001 compared with controls). Four hours (t4): One-way ANOVA (Bonferroni posttests *P < 0.05, **P < 0.01, ***P < 0.001 compared with controls).

No statistically significant differences were found between control and other animals in terms of weight, anesthetic dose, volume of fluid administered, HR, MAP, base deficit, or lactate at baseline (see Additional file 1, Table S1). Compared with sham animals, control animals had a significantly higher HR (*P *< 0.05) at 4 hours and a lower MAP (*P *< 0.01) compared with baseline at this time (see Additional file 2, Table S2). At this point, plasma lactate (*P *< 0.001) and base deficit (*P *< 0.01), neutrophil cell surface CD11b expression (*P *< 0.001), pulmonary tissue MPO activity (*P *< 0.01), and plasma TNF-α (*P *< 0.01), IL-1β (*P *< 0.01), IL-6 (*P *< 0.001), and IL-10 (*P *< 0.05) were also significantly higher in control animals when compared with sham animals (Figures 1, 2, 3, and 4 and Additional file 3, Figure S1). Levels of plasma TNF-α at 1 hour (that is, peak levels) were also significantly higher in control animals (*P *< 0.001).

**Figure 5:**
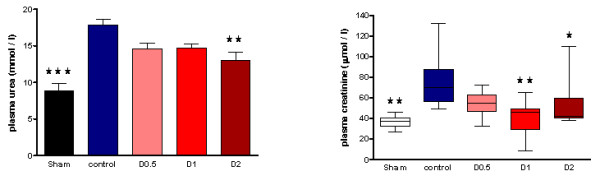
**Plasma urea and creatinine sampled 4 hours after laparotomy and endotoxemia (*n *= 8 all groups), experiment 1**. Smaller increases were observed in pooled dopexamine groups compared with controls (*P *< 0.005). Data presented as mean (SEM) when all groups were normally distributed; otherwise, median (IQR) when more than one group were not normally distributed. Urea, one-way ANOVA (Bonferroni posttests, **P < 0.01, ***P < 0.001 compared with controls). Creatinine: Kruskal-Wallis test (*post hoc *unpaired t tests (sham and D1), **P < 0.01 compared with controls; *post hoc *Mann-Whitney test (D2), *P < 0.05 compared with controls).

**Figure 6:**
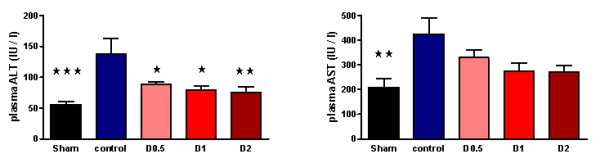
**Plasma alanine aminotransferase (ALT) and aspartate aminotransferase (AST) sampled four hours following laparotomy and endotoxaemia (*n *= 8 all groups), experiment 1**. Smaller increases were observed in pooled dopexamine groups compared with controls (*P *< 0.05). Data presented as mean (SEM). One-way ANOVA (Bonferroni posttests, *P < 0.05, **P < 0.01, ***P < 0.001, compared with controls).

After LPS administration, CD11b expression increased in control animals, whereas CD11a expression decreased (Figure 2 and Additional file 4, Figure S2). Endotoxemia resulted in significant organ injury, as evidenced by control-group plasma urea (*P *< 0.001), creatinine (*P *< 0.001), ALT (*P *< 0.001), and AST (*P *< 0.01) being significantly greater than that of the sham group (Figures 5 and 6).

When compared with control animals, HR was higher in the dopexamine groups at 4 hours after LPS, although this was not statistically significant. Although MAP decreased to a similar extent in controls and all dopexamine groups, MAP was slightly better maintained in D2 animals (Additional file 2, Table S2, and Additional file 5, Figure S3). The increase in plasma lactate was less in dopexamine-treated animals than in control animals (*P *< 0.001 controls versus pooled dopexamine) with corresponding changes in base deficit (*P *< 0.05 controls versus pooled dopexamine) (Figure 1). Compared with control animals, dopexamine at all doses also significantly attenuated the increase in TNF-α (any dose, minimum *P *< 0.05), IL-1β (any dose, minimum *P *< 0.05), and IL-6 (any dose, minimum *P *< 0.01) at 4 hours, whereas the reduction in IL-10 achieved significance only at doses of 0.5 and 1 μg/kg/min (*P *< 0.05 minimum) (Figure 4; Additional file 3, Figure S1). Peak plasma TNF-α was also attenuated at these doses, although significantly in only D2 animals (*P *< 0.05). CD11a expression was unaffected by dopexamine (Additional file 4, Figure S2), but in D1 and D2 groups, dopexamine infusion was associated with significantly decreased CD11b expression at 4 hours (*P *< 0.01 minimum) (Figure 2), as well as significantly reduced pulmonary MPO activity (*P *< 0.05 minimum) (Figure 3). Notably, dopexamine infusion was also associated with significant reductions in renal (*P *< 0.005 pooled dopexamine versus controls) and hepatic injury (*P *< 0.05 pooled dopexamine versus controls) (Figures 5 and 6).

### Experiment 2

**Figure 7:**
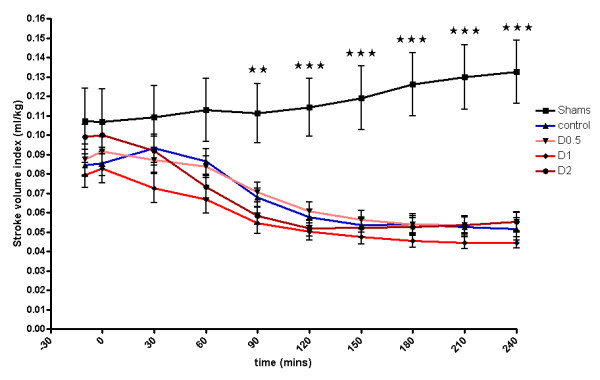
**Stroke volume index 4 hours after laparotomy and endotoxemia, experiment 2**. Stroke volume index decreased significantly throughout the experiment, but no differences were observed between controls and dopexamine-treated animals. Data presented as mean (SEM). Two-way ANOVA (Bonferroni posttests **P < 0.01, ***P < 0.001 sham compared with controls).

**Figure 8:**
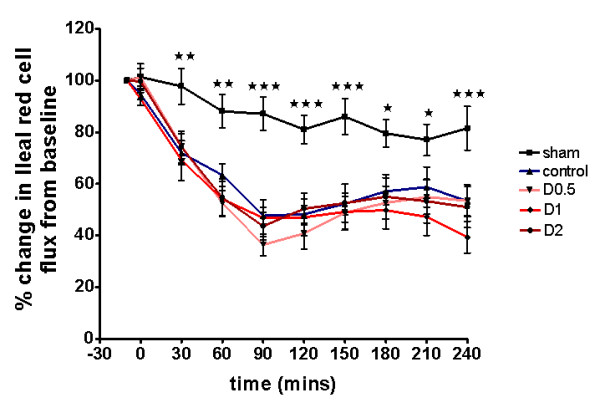
**Regional microvascular flow (ileal red-cell flux) 4 hours after laparotomy and endotoxemia, experiment 2**. Flow decreased significantly throughout the experiment, but no differences were observed between controls and dopexamine-treated animals. Data presented as mean (SEM). Two-way ANOVA (Bonferroni posttests, *P < 0.05, **P < 0.01, ***P < 0.001 sham compared with controls).

No statistically significant differences were found between groups in terms of animal weight, dose of anesthetic administered, or volumes of fluid infused at baseline (see Additional file 6, Table S3). Baseline MAP, HR, CI, SVI, and lactate were not significantly different between groups (see Additional file 7, Table S4; Additional file 8, Figure S4; and Additional file 9, Figure S5; Figure 7). In the sham group, CI and SVI increased progressively (Figure 7 and Additional file 9, Figure S5), whereas ileal flux decreased to a mean of 82% of baseline over a 4-hour period (*P *< 0.05 versus baseline) (Figure 8). In control-group animals, ileal flux also decreased over time, but more rapidly and to levels below those observed in sham animals (*P *< 0.05 minimum, from 30 minutes onward) (Figure 8). This was associated with a moderate but significant decrease in CI over 4 hours (*P *< 0.05 versus baseline) and a more-marked decrease in SVI over the same period (*P *< 0.0005 versus baseline), possibly reflecting the importance of compensatory tachycardia in this model. By 4 hours, control-group plasma lactate (*P *< 0.01) and base deficit (*P *< 0.05) were significantly increased compared with sham animals (Additional file 7, Table S4). With regard to organ dysfunction, endotoxemia in experiment 2 also resulted in organ injury, although the changes did not reach statistical significance for AST (see Additional file 10, Table S5).

The addition of dopexamine at any dose did not significantly affect MAP (Additional file 7, Table S4, and Additional file 8, Figure S4) and did not attenuate the LPS-induced decreases in SVI and CI, despite a significant increase in heart rate (Figure 7; Additional file 9, Figure S5; Additional file 7, Table S4). Moreover, dopexamine did not influence the decrease in ileal red-cell flux (Figure 8), and in contrast to experiment 1, dopexamine at any dose (and when dopexamine data were pooled) was not associated with any significant differences in end-experiment plasma lactate, base deficit, or organ function when compared with controls (Additional file 7, Table S4, and Additional file 10, Table S5), with the exception of ALT in the D2 group (*P *< 0.05 versus controls).

Comparing experiments 1 and 2, groups were similar with respect to baseline characteristics. By design, animals in experiment 2 received more fluid to compensate for the more-sustained and severe surgical insult (Additional file 1, Table S1, and Additional file 6, Table S3). Nevertheless, baseline HR, MAP, and lactate were similar to those of equivalent groups in experiment 1 (Additional file 2, Table S2, and Additional file 7, Table S4). However, an overall reduction in the severity of injury and lactatemia appeared to occur in control animals in experiment 2, as compared with experiment 1 (Additional file 7, Table S4, and Additional file 10, Table S5).

For completeness, standard deviations of data are presented for both experiment 1 (Additional file 11, Table S6) and experiment 2 (Additional file 12, Table S7).

## Discussion

This study showed that, in a rodent model of laparotomy and endotoxemia, dopexamine can attenuate the systemic inflammatory response, limit the degree of lactic acidosis, and protect against organ injury. In the first experiment, dopexamine infusion was associated with a reduced systemic inflammatory response, as evidenced by decreased circulating levels of TNF-α, IL-1β, and IL-6, and decreased leukocyte expression of the cell-adhesion molecule CD11b. In turn, this was associated with reduced pulmonary MPO activity, a marker of pulmonary leukocyte infiltration. Overall, dopexamine-treated animals sustained less organ injury than did control animals. Interestingly, these potentially beneficial effects occurred at doses of dopexamine that had little or no effect on blood pressure and did not alter cardiac output or mesenteric regional microvascular flow in the second experiment.

Based on these findings, we suggest that after a combined surgical and infectious insult, dopexamine can attenuate the increase in circulating levels of inflammatory mediators and reduce leukocyte expression of the cell-adhesion molecule CD11b. As a consequence, leukocyte-endothelial adhesion and transmigration into tissues is decreased, with a reduction in organ injury. This suggestion is consistent with the findings of previous studies indicating that catecholamines can inhibit cytokine release [16,22]. Although not intrinsically chemotactic, TNF-α and IL-1β upregulate endothelial and leukocyte expression of adhesion molecules [23,24], and hence promote leukocyte-endothelial adhesion and migration into tissues. Previous work also showed that adrenergic agents can decrease leukocyte expression of adhesion molecules [25], whereas *in vivo *microscopy suggests that dopexamine may decrease leukocyte-endothelial adhesion in the mesenteric circulation [16,17]. Dopexamine has been shown to decrease free radical-mediated tissue injury in other animal models [26,27], whereas the β2-agonist terbutaline reduced nitric oxide and superoxide levels in endotoxemic rats [28]. These findings are consistent with our current understanding of the important role played by endogenous catecholamines in the regulation of immune cell function [15].

In contrast to our recent clinical findings in high-risk surgery patients [6], in the present study, these effects were observed at doses of dopexamine that had little or no effect on arterial pressure and did not alter cardiac output or mesenteric regional microvascular flow, suggesting that dopexamine has independent antiinflammatory actions that can reduce inflammation and organ injury in this model.

Our findings appear to contrast with those of a recent small clinical trial performed by our group, which identified significant increases in tissue microvascular flow and oxygenation but no changes in inflammatory mediators after major surgery [6]. Several differences may explain this. First, the time points at which serum was collected in the clinical trial were not suitable for the identification of a modified inflammatory response in dopexamine-treated subjects. Second, differences in organ injury are more readily identified in previously healthy rats than in patients with significant comorbid disease. Notwithstanding these differences, the findings of the clinical trial do not contradict those of this more recent laboratory work.

A number of limitations complicate the interpretation of these experiments. Although this model is one of combined laparotomy and sepsis, it is impossible to evaluate the effects of the surgery alone from the design, and differences between groups are related to the effects of treatment on sepsis, albeit in the setting of abdominal surgery. Although the models used for the two experiments were similar, we cannot be certain that the additional surgical preparation required for experiment 2 did not cause hemodynamic differences between the two preparations (for example, in regional microvascular flow). However, the fact that values for HR, blood pressure, and lactate at baseline and throughout the 4-hour experimental period were similar argues against this possibility. It would have been preferable to perform all measurements in one experiment rather than two, but as experiment 1 required 1.2 ml of blood (5% of the blood volume of a 300-g rat) to be intermittently sampled before 4 hours, and laser Doppler flowmetry is exquisitely sensitive to even minor changes in circulating volume, it was thought that a separate experiment should be performed to assess independently the effects of dopexamine and fluid on microvascular flow in this setting.

It is also reassuring that many of the findings from the current experiments are consistent with those of previous work, such as the reduction in regional microvascular flux seen with LPS, as well as the amelioration of renal dysfunction and downregulation of CD11b integrins caused by dopexamine [16,25,29]. Many methods have been used to evaluate the microcirculation *in vivo*, each of which has limitations. Laser Doppler flowmetry, as used here, may lack the sensitivity required to detect small changes in flow heterogeneity of microvascular flow. These changes may have been better detected by other means of monitoring the microcirculation (for example, intravital microscopy). Moreover deterioration in the quality of ileal preparations over time is a recognized phenomenon in microvascular studies involving bowel exteriorization.

The failure to demonstrate improvements in regional microvascular flow in experiment 2 might also be explained by other differences between our model and those in which dopexamine was shown to improve it. These include but are not limited to endotoxin serotype and fluid regimens [16,17]. The limited protective effect of dopexamine in terms of organ injury might then reflect the absence of improvements in regional microvascular flow. It is also possible that the observed reduction in lactic acidosis was attributable to direct cellular or metabolic mechanisms rather than being secondary to improved tissue oxygenation [30,31].

## Conclusions

In summary, these findings suggest that, in a rodent model of laparotomy and endotoxemia, dopexamine at doses within the clinical range can attenuate TNF-α release, tissue leukocyte infiltration, and hence organ injury at doses that do not alter global hemodynamics or regional microvascular flow. These findings support the suggestion that the immunomodulatory effects of catecholamines are likely to be clinically significant when these agents are used in critically ill surgical patients. Further research is required to explore the potential benefits and possible dangers of immune modulation induced by adrenergic agents in clinical practice.

## Key messages

• In a resuscitated model of normotensive sepsis and surgical stress, the addition of clinically relevant doses of dopexamine can attenuate the inflammatory response and ameliorate organ dysfunction.

• This occurs at doses at which dopexamine appears to have little additional effect on cardiac index, stroke volume index, or regional microvascular flow.

## Abbreviations

ALT: alanine aminotransferase; AST: aspartate aminotransferase; CI: cardiac index; HR: heart rate; IL-1β/6/10: interleukin 1 beta/6/10; LPS: lipopolysaccharide; MAP: mean arterial pressure; MFI: mean fluorescent intensity; MPO: myeloperoxidase; SVI: stroke volume index; TNF-α: tumor necrosis factor alpha.

## Competing interests

MNB, NSAP, EB, and MC have no interests to declare. RP and CJH are named inventors on a lapsed patent application relating to a specific use for dopexamine. CJH is a member of the editorial board for the journal *Shock*. CT is CEO of William Harvey Research Limited, which is a CRO and has conducted contracted research in the area of critical care. CT is also European Editor for the journal *Shock*.

## Authors' contributions

MNB carried out the *in vivo *studies, cytokine analyses, flow-cytometry studies, and statistical analysis. NSP provided invaluable support during flow cytometry and *in vivo *studies, in addition to providing advice on statistical analysis and study design. EB and MC performed MPO analysis. CT participated in the design and coordination of the study and provided guidance throughout. RMP conceived of the study, participated in its design and coordination, and helped with statistical analysis. RMP, CJH, CT, and MNB together drafted the manuscript. All authors read and approved the final manuscript.

## Supplementary Material

Additional file 1 **Table S1. Baseline characteristics for experiment 1 (*n *= 8 all groups)**. Data presented as mean (SEM) when all groups were normally distributed; otherwise, median (IQR) if more than one group were not normally distributed. Click here for file

Additional file 2 **Table S2. Hemodynamic parameters, end-of-experiment lactate and arterial blood gas data for experiment 1**. All groups hemodynamics and lactate, arterial blood gas data (pH, base deficit, P_a_CO_2_, and P_a_O_2_) *n *= 8 (except D0.5 and D1 ABG data, *n *= 6; Control P_a_CO_2 _and P_a_O_2 _data only, *n *= 6). Data presented as mean (SEM) when all groups were normally distributed; otherwise, median (IQR) if more than one group were not normally distributed. One-way ANOVA (Bonferroni posttests, *P < 0.05, **P < 0.01, ***P < 0.001 versus controls). Final HR only: Kruskal-Wallis tests (*post hoc *Mann-Whitney tests, *P < 0.05 versus controls). Click here for file

Additional file 3 **Figure S1. Plasma cytokine concentrations after laparotomy and endotoxemia. (IL-1β: *n *= 8 sham and control, *n *= 7 D0.5, *n *= 6 D1, *n *= 4 D2; IL-6: all *n *= 8 except *n *= 7 control and D2; IL-10: *n *= 8 sham and control, *n *= 7 D0.5 and D1, *n *= 6 D2), experiment 1**. Dopexamine was associated with smaller increases in all groups compared with controls. Data presented as mean (SEM). One-way ANOVA (Bonferroni posttests, *P < 0.05, **P < 0.01, ***P < 0.001 compared with controls). Click here for file

Additional file 4 **Figure S2. Circulating neutrophil CD11a mean fluorescent intensity (MFI) at baseline and 4 hours after laparotomy and endotoxemia (*n *= 7 D0.5; *n *= 8 all others), experiment 1**. Data presented as mean (SEM). Two-way ANOVA (Bonferroni posttests, *P < 0.05 compared with controls). Click here for file

Additional file 5 **Figure S3. Mean arterial pressure for all groups (experiment 1) 4 hours after laparotomy and endotoxemia (*n *= 8 all groups)**. Data presented as mean (SEM). Click here for file

Additional file 6 **Table S3. Baseline characteristics for experiment 2 (*n *= 8 all groups)**. Data presented as mean (SEM) when all groups were normally distributed; otherwise, median (IQR) if more than one group were not normally distributed. Click here for file

Additional file 7 **Table S4. Hemodynamic parameters, end-of-experiment lactate and arterial blood gas data for experiment 2**. All groups hemodynamics, lactate, and arterial blood gas data (pH, base deficit, P_a_CO_2_, and P_a_O_2_) *n *= 8 (except D2, ABG data *n *= 7). Data presented as mean (SEM). One-way ANOVA (Bonferroni posttests *P < 0.05, **P < 0.01, ***P < 0.001 versus controls). Click here for file

Additional file 8 **Figure S4. Mean arterial pressure for all groups (experiment 2) 4 hours after laparotomy and endotoxemia (*n *= 8 all groups)**. Data presented as mean (SEM). Click here for file

Additional file 9 **Figure S5. Relative cardiac index for all groups (experiment 2) 4 hours after laparotomy and endotoxemia (*n *= 8 all groups)**. Data presented as mean (SEM). Two-way ANOVA (Bonferroni posttests, *P < 0.05, **P < 0.01, ***P < 0.001 compared with controls). Click here for file

Additional file 10 **Table S5. Plasma urea, creatinine, alanine aminotransferase (ALT), and aspartate aminotransferase (AST) sampled 4 hours after laparotomy and endotoxemia in experiment 2 (all *n *= 8)**. Data presented as mean (SEM) when all groups were normally distributed; otherwise, median (IQR) if more than one group was not normally distributed. Kruskal-Wallis test (urea and ALT: *post hoc *Mann-Whitney test *P < 0.05, **P < 0.005, ***P < 0.001 versus controls). Kruskal-Wallis test (creatinine: *post hoc *Unpaired *t *test, ***P < 0.001 versus controls). Click here for file

Additional file 11 **Table S6. Standard deviations of all data presented in tables for experiment 1**. Click here for file

Additional file 12 **Table S7. Standard deviations of all data presented in tables for experiment 2**. Click here for file
