# Supplementary material for: Dopexamine can attenuate the inflammatory response and protect against organ injury in the absence of significant effects on hemodynamics or regional microvascular flow
Source: Crit Care. 2013 Mar 28;17(2):R57. doi: 10.1186/cc12585 (PMC3672538; doi:10.1186/cc12585)
Supplement: Additional file 1 — Table S1. Baseline characteristics for experiment 1 (n = 8 all groups). Data presented as mean (SEM) when all groups were normally distributed; otherwise, median (IQR) if more than one group were not normally distributed. [file cc12585-S1.DOC]

|  | **Experiment 1** | | | | |
| --- | --- | --- | --- | --- | --- |
|  | ***Sham*** | ***Control*** | ***D 0.5*** | ***D1*** | ***D2*** |
| Weight (g) | 300 (7) | 304 (18) | 325 (16) | 260 (15) | 320 (17) |
| Fluid (ml kg^-1^) | 21.6  (21.5 – 21.7) | 21.4  (21.0 – 22.6) | 22.9  (22.2 – 23.5) | 22.8  (22.3 – 22.8) | 22.1  (21.7 – 23.5) |
| Thiopental  (mg kg^-1^) | 194.5 (8.6) | 163.1 (10.7) | 160.5 (11.3) | 161.5 (10.5) | 158.8 (8.3) |
