# Supplementary material for: Dopexamine can attenuate the inflammatory response and protect against organ injury in the absence of significant effects on hemodynamics or regional microvascular flow
Source: Crit Care. 2013 Mar 28;17(2):R57. doi: 10.1186/cc12585 (PMC3672538; doi:10.1186/cc12585)
Supplement: Additional file 2 — Table S2. Hemodynamic parameters, end-of-experiment lactate and arterial blood gas data for experiment 1. All groups hemodynamics and lactate, arterial blood gas data (pH, base deficit, PaCO2, and PaO2) n = 8 (except D0.5 and D1 ABG data, n = 6; Control PaCO2 and PaO2 data only, n = 6). Data presented as mean (SEM) when all groups were normally distributed; otherwise, median (IQR) if more than one group were not normally distributed. One-way ANOVA (Bonferroni posttests, *P < 0.05, **P < 0.01, ***P < 0.001 versus controls). Final HR only: Kruskal-Wallis tests (post hoc Mann-Whitney tests, *P < 0.05 versus controls). [file cc12585-S2.DOC]

|  | **Experiment 1** | | | | |
| --- | --- | --- | --- | --- | --- |
|  | ***Sham*** | ***Control*** | ***D 0.5*** | ***D1*** | ***D2*** |
| Initial HR (bpm) | 386 (9) | 402 (12) | 400 (20) | 393 (12) | 367 (27) |
| Final HR (bpm) | **415**  **(410 - 427)*** | **467**  **(438 - 492)** | **504**  **(472 - 515)** | **484**  **(481 - 496)** | **485**  **(472 - 492)** |
| Initial MAP (mmHg) | 97 (5) | 110 (6) | 108 (4) | 103 (4) | 115 (5) |
| Final MAP (mmHg) | 96 (4) | 85 (7) | 94 (4) | 93 (7) | 104 (5) |
| End experiment lactate  (mmol l^-1^) | 1.3 (0.2)*** | 4.0 (0.5) | 2.6 (0.4) | 2.0 (0.4)** | 2.1 (0.3)** |
| End experiment base  deficit (mmol l^-1^) | 1.0 (1.2)** | 7.9 (2.2) | 5.6 (1.0) | 2.2 (1.4)* | 2.9 (0.9) |
| End experiment pH | 7.43 (0.01) | 7.33 (0.06) | 7.41 (0.02) | 7.38 (0.02) | 7.45 (0.04) |
| End experiment P_a_CO_2_ (kPa) | 4.5 (0.2) | 3.7 (0.4) | 3.8 (0.3) | 5.0 (0.3) | 3.9 (0.4) |
| End experiment P_a_O_2_ (kPa) | 11.5 (0.5) | 12.5 (0.6) | 11.6 (0.9) | 10.7 (0.6) | 12.0 (0.7) |
