# Supplementary figures and images for: Dopexamine can attenuate the inflammatory response and protect against organ injury in the absence of significant effects on hemodynamics or regional microvascular flow
Source: Crit Care. 2013 Mar 28;17(2):R57. doi: 10.1186/cc12585 (PMC3672538; doi:10.1186/cc12585)

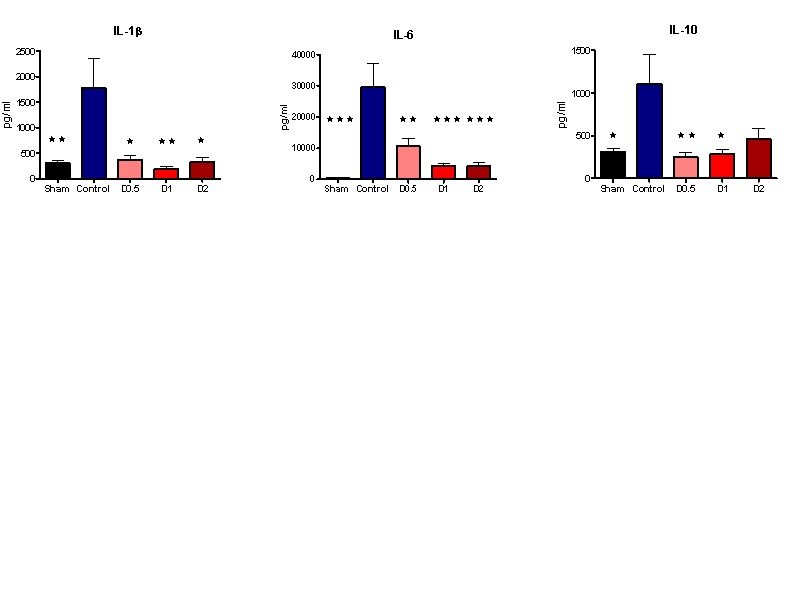

Supplement: Additional file 3 — Figure S1. Plasma cytokine concentrations after laparotomy and endotoxemia. (IL-1β: n = 8 sham and control, n = 7 D0.5, n = 6 D1, n = 4 D2; IL-6: all n = 8 except n = 7 control and D2; IL-10: n = 8 sham and control, n = 7 D0.5 and D1, n = 6 D2), experiment 1. Dopexamine was associated with smaller increases in all groups compared with controls. Data presented as mean (SEM). One-way ANOVA (Bonferroni posttests, *P < 0.05, **P < 0.01, ***P < 0.001 compared with controls). [file cc12585-S3.JPEG]

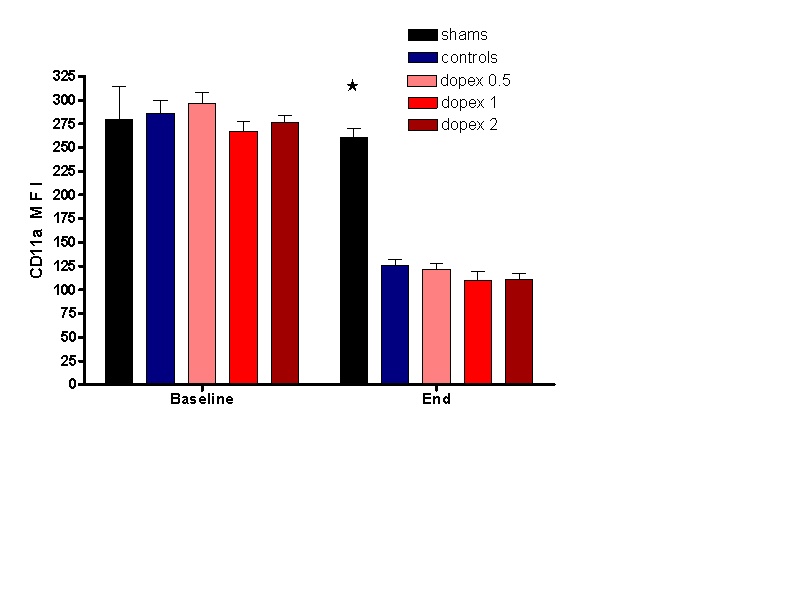

Supplement: Additional file 4 — Figure S2. Circulating neutrophil CD11a mean fluorescent intensity (MFI) at baseline and 4 hours after laparotomy and endotoxemia (n = 7 D0.5; n = 8 all others), experiment 1. Data presented as mean (SEM). Two-way ANOVA (Bonferroni posttests, *P < 0.05 compared with controls). [file cc12585-S4.JPEG]

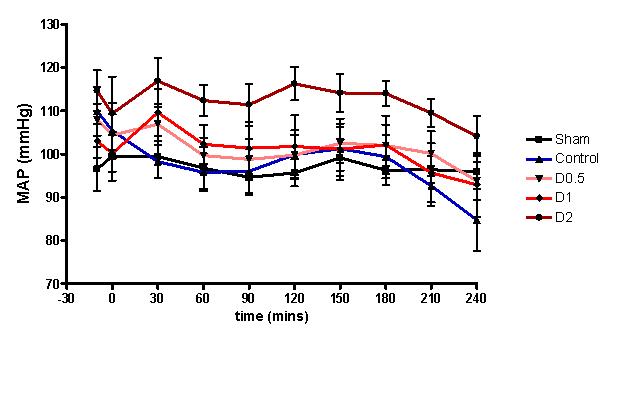

Supplement: Additional file 5 — Figure S3. Mean arterial pressure for all groups (experiment 1) 4 hours after laparotomy and endotoxemia (n = 8 all groups). Data presented as mean (SEM). [file cc12585-S5.JPEG]

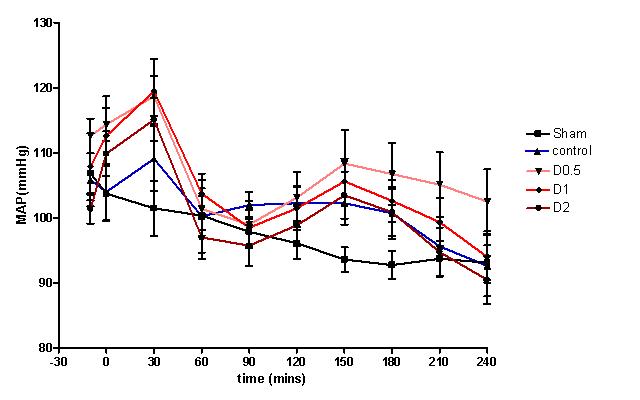

Supplement: Additional file 8 — Figure S4. Mean arterial pressure for all groups (experiment 2) 4 hours after laparotomy and endotoxemia (n = 8 all groups). Data presented as mean (SEM). [file cc12585-S8.JPEG]

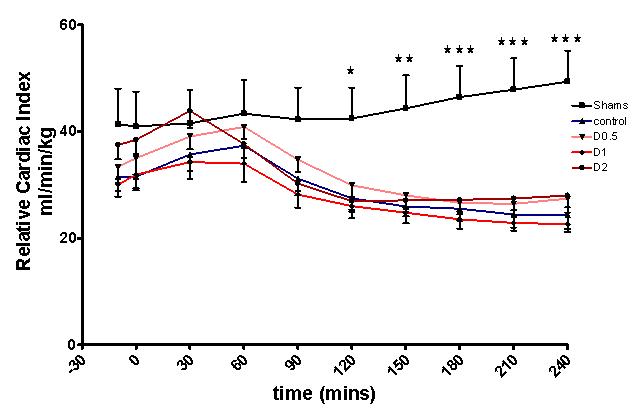

Supplement: Additional file 9 — Figure S5. Relative cardiac index for all groups (experiment 2) 4 hours after laparotomy and endotoxemia (n = 8 all groups). Data presented as mean (SEM). Two-way ANOVA (Bonferroni posttests, *P < 0.05, **P < 0.01, ***P < 0.001 compared with controls). [file cc12585-S9.JPEG]
