# Supplementary material for: Dopexamine can attenuate the inflammatory response and protect against organ injury in the absence of significant effects on hemodynamics or regional microvascular flow
Source: Crit Care. 2013 Mar 28;17(2):R57. doi: 10.1186/cc12585 (PMC3672538; doi:10.1186/cc12585)
Supplement: Additional file 6 — Table S3. Baseline characteristics for experiment 2 (n = 8 all groups). Data presented as mean (SEM) when all groups were normally distributed; otherwise, median (IQR) if more than one group were not normally distributed. [file cc12585-S6.DOC]

|  | **Experiment 2** | | | | |
| --- | --- | --- | --- | --- | --- |
|  | ***Sham*** | ***Control*** | ***D 0.5*** | ***D1*** | ***D2*** |
| Weight (g) | 275  (265 – 305) | 320  (290 – 320) | 320  (285 – 325) | 305  (290 – 320) | 300  (280 – 300) |
| Fluid (ml kg^-1^) | 24.3  (23.8 – 24.3) | 23.8  (23.8 – 24.0) | 23.9  (23.7 – 24.2) | 23.8  (23.5 – 23.9) | 24.0  (23.8 – 24.1) |
| Thiopental  (mg kg^-1^) | 172.8 (3.2) | 156.5 (7.6) | 160 (9.3) | 152 (6) | 157.2 (4.3) |
