# Supplementary material for: Dopexamine can attenuate the inflammatory response and protect against organ injury in the absence of significant effects on hemodynamics or regional microvascular flow
Source: Crit Care. 2013 Mar 28;17(2):R57. doi: 10.1186/cc12585 (PMC3672538; doi:10.1186/cc12585)
Supplement: Additional file 7 — Table S4. Hemodynamic parameters, end-of-experiment lactate and arterial blood gas data for experiment 2. All groups hemodynamics, lactate, and arterial blood gas data (pH, base deficit, PaCO2, and PaO2) n = 8 (except D2, ABG data n = 7). Data presented as mean (SEM). One-way ANOVA (Bonferroni posttests *P < 0.05, **P < 0.01, ***P < 0.001 versus controls). [file cc12585-S7.DOC]

|  | **Experiment 2** | | | | |
| --- | --- | --- | --- | --- | --- |
|  | ***Sham*** | ***Control*** | ***D 0.5*** | ***D1*** | ***D2*** |
| Initial HR (bpm) | 386 (10) | 373 (13) | 383 (7) | 381 (11) | 378 (10) |
| Final HR (bpm) | 373 (8)* | 473 (10) | 497 (7) | 507 (4)* | 507 (7)* |
| Initial MAP (mmHg) | 107 (3) | 106 (4) | 113 (3) | 108 (5) | 101 (2) |
| Final MAP (mmHg) | 93 (3) | 93 (5) | 103 (5) | 94 (4) | 91 (4) |
| End experiment lactate  (mmol l^-1^) | 1.7 (0.2)** | 3.7 (0.4) | 2.9 (0.3) | 3.6 (0.4) | 3.3 (0.4) |
| End experiment base  deficit (mmol l^-1^) | -1.5 (0.5)* | 9.0 (1.4) | 6.1 (1.1) | 7.7 (0.9) | 8.3 (0.9) |
| End experiment pH | 7.35 (0.01) | 7.34 (0.02) | 7.40 (0.01)* | 7.35 (0.01) | 7.36 (0.01) |
| End experiment P_a_CO_2_ (kPa) | 6.8 (0.2)  *** | 3.8 (0.3) | 3.8 (0.3) | 4.1 (0.2) | 3.8 (0.2) |
| End experiment P_a_O_2_ (kPa) | 10.1 (0.5) *** | 13.9 (0.7) | 13.3 (0.7) | 13.1 (0.5) | 13.7 (0.8) |
