# Supplementary material for: Dopexamine can attenuate the inflammatory response and protect against organ injury in the absence of significant effects on hemodynamics or regional microvascular flow
Source: Crit Care. 2013 Mar 28;17(2):R57. doi: 10.1186/cc12585 (PMC3672538; doi:10.1186/cc12585)
Supplement: Additional file 10 — Table S5. Plasma urea, creatinine, alanine aminotransferase (ALT), and aspartate aminotransferase (AST) sampled 4 hours after laparotomy and endotoxemia in experiment 2 (all n = 8). Data presented as mean (SEM) when all groups were normally distributed; otherwise, median (IQR) if more than one group was not normally distributed. Kruskal-Wallis test (urea and ALT: post hoc Mann-Whitney test *P < 0.05, **P < 0.005, ***P < 0.001 versus controls). Kruskal-Wallis test (creatinine: post hoc Unpaired t test, ***P < 0.001 versus controls). [file cc12585-S10.DOC]

|  | ***Sham*** | ***Control*** | ***D 0.5*** | ***D1*** | ***D2*** |
| --- | --- | --- | --- | --- | --- |
| Urea (mmol l^-1^) | 10.0  (9.4 – 10.2)*** | 17.3  (16.4 – 18.4) | 15.9  (15.1 – 19.1) | 18.7  (16.6 – 19.3) | 18.6  (18.2 – 19.6) |
| Creatinine  (μmol l^-1^) | 34.8  (33.0 – 39.9)*** | 57.9  (52.0 – 73.4) | 52.6  (45.7 – 54.2) | 61.8  (56.3 – 71.5) | 62.6  (59.4 – 66.7) |
| ALT (IU l^-1^) | 54.4  (48.0 – 59.8)** | 81.8  (73.5 – 85.3) | 78.9  (65.6 – 99.5) | 79.0  (62.3 – 107) | 61.4  (58.0 – 72.7)* |
| AST (IU l^-1^) | 267 (38) | 337 (30) | 335 (43) | 383 (52) | 377 (38) |
