# Supplementary material for: Dopexamine can attenuate the inflammatory response and protect against organ injury in the absence of significant effects on hemodynamics or regional microvascular flow
Source: Crit Care. 2013 Mar 28;17(2):R57. doi: 10.1186/cc12585 (PMC3672538; doi:10.1186/cc12585)
Supplement: Additional file 11 — Table S6. Standard deviations of all data presented in tables for experiment 1. [file cc12585-S11.DOC]

|  | ***Sham*** | ***Control*** | ***D 0.5*** | ***D1*** | ***D2*** |
| --- | --- | --- | --- | --- | --- |
| Weight (g) | 19 | 50 | 46 | 43 | 48 |
| Fluid (ml kg^-1^) | 1.0 | 1.3 | 1.0 | 0.4 | 1.3 |
| Thiopental  (mg kg^-1^) | 24.4 | 30.3 | 32.0 | 29.6 | 23.5 |
| Initial HR (bpm) | 25 | 35 | 55 | 33 | 75 |
| Final HR (bpm) | 25 | 35 | 39 | 16 | 12 |
| Initial MAP (mmHg) | 14 | 16 | 11 | 11 | 13 |
| Final MAP (mmHg) | 11 | 12 | 12 | 21 | 13 |
| End experiment lactate  (mmol l^-1^) | 0.7 | 1.5 | 1.0 | 1.1 | 0.9 |
| End experiment base deficit  (mmol l^-1^) | 3.4 | 6.2 | 2.4 | 3.4 | 2.4 |
| End experiment pH | 0.04 | 0.16 | 0.04 | 0.06 | 0.1 |
| End experiment P_a_CO_2_ (kPa) | 0.6 | 0.9 | 0.6 | 0.7 | 1.1 |
| End experiment P_a_O_2_ (kPa) | 1.5 | 1.5 | 2.2 | 1.4 | 1.9 |
| Urea  (mmol l^-1^) | 2.9 | 2.5 | 2.0 | 1.8 | 2.9 |
| Creatinine (μmol l^-1^) | 6.2 | 27.6 | 12.7 | 17.8 | 24.6 |
| ALT (IU l^-1^) | 17.7 | 71.9 | 13.5 | 19.1 | 23.6 |
| AST (IU l^-1^) | 97 | 195 | 81 | 88 | 78 |
