# Supplementary material for: Dopexamine can attenuate the inflammatory response and protect against organ injury in the absence of significant effects on hemodynamics or regional microvascular flow
Source: Crit Care. 2013 Mar 28;17(2):R57. doi: 10.1186/cc12585 (PMC3672538; doi:10.1186/cc12585)
Supplement: Additional file 12 — Table S7. Standard deviations of all data presented in tables for experiment 2. [file cc12585-S12.DOC]

|  | ***Sham*** | ***Control*** | ***D 0.5*** | ***D1*** | ***D2*** |
| --- | --- | --- | --- | --- | --- |
| Weight (g) | 26 | 39 | 26 | 21 | 12 |
| Fluid (ml kg^-1^) | 0.3 | 0.2 | 0.3 | 0.3 | 0.2 |
| Thiopental  (mg kg^-1^) | 8.9 | 21.4 | 26.3 | 17.1 | 12.2 |
| Initial HR (bpm) | 30 | 37 | 20 | 33 | 27 |
| Final HR (bpm) | 23 | 27 | 21 | 12 | 19 |
| Initial MAP (mmHg) | 12 | 12 | 8 | 15 | 6 |
| Final MAP (mmHg) | 8 | 13 | 14 | 11 | 10 |
| End experiment lactate  (mmol l^-1^) | 0.5 | 1.0 | 0.8 | 1.2 | 1.0 |
| End experiment base deficit  (mmol l^-1^) | 1.3 | 4.0 | 3.0 | 2.7 | 2.3 |
| End experiment pH | 0.03 | 0.05 | 0.03 | 0.04 | 0.03 |
| End experiment P_a_CO_2_ (kPa) | 0.7 | 0.8 | 0.9 | 0.6 | 0.6 |
| End experiment P_a_O_2_ (kPa) | 1.3 | 2.1 | 2.0 | 1.3 | 2.2 |
| Urea  (mmol l^-1^) | 2.0 | 2.0 | 3.0 | 1.8 | 1.3 |
| Creatinine (μmol l^-1^) | 4.3 | 12.6 | 13.9 | 14.8 | 7.3 |
| ALT (IU l^-1^) | 7.5 | 19.0 | 23.4 | 42.0 | 55.1 |
| AST (IU l^-1^) | 106 | 86 | 122 | 146 | 108 |
